# “I Trust in Staff's Creativity”—The Impact of COVID-19 Lockdowns on Physical Activity Promotion in Nursing Homes Through the Lenses of Organizational Sociology

**DOI:** 10.3389/fspor.2020.589214

**Published:** 2020-10-07

**Authors:** Annika Frahsa, Dorothee Altmeier, Jannika M. John, Hannes Gropper, Hanna Granz, Rebekka Pomiersky, Daniel Haigis, Gerhard W. Eschweiler, Andreas M. Nieß, Gorden Sudeck, Ansgar Thiel

**Affiliations:** ^1^Institute of Sport Science, Eberhard Karls University Tübingen, Tübingen, Germany; ^2^Interfaculty Research Institute for Sport and Physical Activity, Eberhard Karls University Tübingen, Tübingen, Germany; ^3^Department of Sports Medicine, University Hospital Tübingen, Tübingen, Germany; ^4^Centre for Geriatric Medicine, University Hospital Tübingen, Tübingen, Germany

**Keywords:** organizational theory, organizational culture, long-term care, COVID-19, physical activity, daily activity

## Abstract

**Objectives:** The health-enhancing benefits or regular physical activity (PA) reach into old age. With the emergence of the coronavirus disease of 2019 (COVID-19) pandemic and the associated national lockdowns and restrictions, nursing home residents were restrained from being physically active. In our study, we aimed to assess the impact of the COVID-19-related restrictions on PA promotion in nursing homes from an organizational-sociological lens.

**Methods:** We collected data in eight nursing homes in Germany. Data collection included (i) semistructured interviews focusing on COVID-19-related restrictions and their effects on nursing homes from the home administrators' perspectives; (ii) open-ended surveys with nursing home staff and relatives focusing on daily routines and contact restrictions; and (iii) collection of documents such as care concepts, mission statements, and weekly activity plans. We analyzed all data with a reflexive thematic analysis approach.

**Results:** We identified three stages of COVID-19-related changes in nursing homes that impacted PA promotion, as follows: (1) external closure and search for emergency control, (2) organizational adaptations to create a livable daily life in the internal environment, and (3) slow reintegration of interactions with the external organizational environment. Document analysis revealed that PA promotion was not part of decision programs or internal staff work descriptions. Rather, PA promotion was delegated to external service providers. The assignment of PA promotion to external providers was not structurally anchored in decision programs, which makes PA promotion not sustainable, particularly during unforeseen events that limit access to the organization. During the pandemic, executive staff believed in internal staff to buffer competencies with regard to PA promotion. Thus, executive staff often considered PA promotion relevant, even during the pandemic, but thought that PA promotion is a task that can be fulfilled by unqualified but motivated internal staff.

**Conclusion:** While our study participants showed a high level of coping-capacity belief, it remains unclear which long-term impacts of COVID-19 on PA promotion in nursing homes are to be expected. At the practice level, executive staff in nursing homes that aim to promote PA within their organization should become aware that PA promotion needs to be incorporated into organizational structures to be implemented and continued in challenging times such as in a pandemic.

## Introduction

### Background

Physical activity (PA) has widely been acknowledged as a decisive behavior to promote health. The health-enhancing benefits of regular PA reach into old age; even small doses of activity can bear positive effects. Despite being a substantial contributor to biopsychosocial well-being and healthy aging, PA tends to be limited in institutions for older people: nursing home residents show low levels of PA and a high prevalence of sedentariness (Barber et al., 2015; Lotvonen et al., 2017). The lack of PA increases the risks of muscle atrophy (Gennuso et al., 2013), falling (Bean et al., 2002), depression (Lampinen et al., 2000), social isolation (Shankar et al., 2011), and, thus, premature mortality.

With the emergence of the coronavirus disease of 2019 (COVID-19) pandemic in the early spring of 2020 and the associated national lockdowns and restrictions, nursing home residents were restrained from being physically active. Worldwide, public health authorities established behavioral guidelines for nursing homes on regional, state, and/or federal levels. By doing so, authorities aimed to protect older people, especially those with chronic conditions and comorbidities, and to decrease the COVID-19-related mortality among those (Dichter et al., 2020; Fallon et al., 2020). In addition, nursing homes tended to have high transmission rates due to their infrastructural and organizational set-ups, such as shared bathroom facilities and common living areas and low readiness when it comes to infection control measures (Davidson and Szanton, 2020). In order to decrease the risk of infection and to protect residents, social and environmental regulations were implemented in several countries worldwide, including the lockdown of nursing homes for both visitors and residents.

This paper will analyze the impact of COVID-19 on PA promotion in a sample of nursing homes in the German state of Baden-Württemberg. In the state of Baden-Württemberg, the lockdown lasted from mid-March to the beginning of May 2020 (State Government of Baden-Württemberg, 2020a; 2020b). The lockdown included the prohibition of visits by relatives and service providers (e.g., hairdressers, physiotherapists, pastoral workers, etc.), restrictions to leave the institution (except for urgent doctor's appointments), and the limitation of group activities as well as everyday activities such as walking the floors or going for a walk outside of the home (State Government of Baden-Württemberg, 2020c).

Although such restrictions aim to protect those at the highest risk for severe illness resulting from the new virus, the restrictions also bear potential health risks through reinforcing isolation, physical inactivity, and sedentary behavior. When staff shield residents in their rooms, they also restrict residents' autonomy and impose mental and physical harms (Gordon et al., 2020). Measures of isolation may be exceptionally challenging for residents with cognitive impairments such as dementia or walking with purpose (Fallon et al., 2020; Gordon et al., 2020). Consequently, Dichter et al. (2020) argue for providing residents with opportunities for walking and spending time outdoors as well as meeting, i.e., seeing and talking, with relatives in accordance with infection management regulations.

The executive and nursing staff play a major role when deciding on the opportunities for residents to be physically active during the lockdown, because the staff are responsible for their protection. To which extent the nursing staff can contribute to the physical activation of residents in times of COVID-19-related restrictions, is unknown by now. The nursing staff are in the epicenter of the pandemic and must put political regulations into practice. On the one hand, nurses have to adapt to changes in care practices and develop hygiene concepts. On the other hand, they are affected by the pandemic themselves: while nursing practices have become increasingly difficult due to the restrictions, institutions are understaffed due to quarantine and staff's sick leaves (Fallon et al., 2020; Oliver, 2020). Executive staff perceive an increase in challenges and concerns during the COVID-19 pandemic, as recent data shows (Hower et al., 2020). Executive staff are concerned about potential infections of residents and staff, insecure about how to proceed in case of an infection, how to acquire protective gear such as masks or cloths, and, not least, how to deal with the increased workload due to hygiene-related education or reduced support from relatives (Hower et al., 2020). Hence, the problems of decreased PA opportunities for residents are probably not very high on management's priority list.

By now, researchers have not yet collected data on the impacts of the COVID-19-related restrictions on PA promotion in nursing homes. Due to the assumption that such impacts concern the entire organization, we take a broader perspective in our research, beyond individual attitudes and experiences. For our perspective, we use an organizational theoretical lens (Lewin, 1951; Luhmann, 2000; Schein, 2017) to assess the impact of COVID-19 lockdowns on PA promotion. This lens allows us to look at the organizational set-up of nursing homes when it comes to not only PA promotion in general but also to PA promotion in times of the COVID-19 pandemic.

### Theoretical Framework

From the perspective of the sociology of organization, the analytical focus is not on the motives or attitudes of people who are employed in the organization (in our study management or caregivers) or use its services (residents or relatives). The organizational sociological focus rather is on the analysis of decision-making on the basis of institutional arrangements, such as collective values, routines of action, and work roles, and also conceptual beliefs or cultural systems (cf. Powell and Dimaggio, 1991; Colignon, 2007). From a system-theoretical perspective, “organizations emerge and (…) reproduce (themselves) when decisions are communicated (…). Everything else–goals, hierarchies, opportunities for rationality, members bound by instructions, or whatever else has been regarded as a criterion of organization–is secondary and can be regarded as the result of the decision-making operations of the system” (Luhmann, 2000 p. 63). Decisions in organizations are based on the so-called decision premises, i.e., specific basic decisions of the organization about subsequent decisions. They form the structure of the organization and give direction to further decisions (cf. Luhmann, 2000 p. 86). Luhmann differentiates three types of decision premises: decision programs (purpose and conditional programs), communication means^1^, and personnel decisions.

Purpose programs are directed toward the purpose of the organization and describe specific future expectations (e.g., differentiated into short- and long-term goals), the fulfillment of which requires the use of certain means (ideally differentiated in orientation toward alternative scenarios). With regard to PA promotion in nursing homes, purpose programs guarantee a sustainable anchoring of PA offers in the organization. If organizational statues do not include PA promotion as a goal, it is ultimately left to the commitment of individuals whether PA takes place or not (cf. Thiel and Meier, 2004; Rütten et al., 2009).

Conditional programs are if-then specifications that are stored in the organization mostly in written form, usually *via* statutes or protocols (Luhmann, 2000, p. 275). An organization's conditional programs stipulate that the occurrence of a certain, predetermined event requires a further decision. For our analysis, conditional programs are important in such a way that, ideally, for certain events, such as a pandemic, certain procedures for how to act are stipulated. Hence, if there are no guidelines on how to deal with PA promotion in the event of a lockdown in nursing homes, then the promotion of PA during this period is not guaranteed (even if it is written down as an organizational goal), but rather inevitably depends on the commitment of individuals.

Communication means in organizations can be described as a system of specification and distribution of organizational tasks. In such a network, competencies that ensure the acceptance of decisions are mutually coupled in both horizontal and vertical directions (Luhmann, 2000). The professional and hierarchical competencies that can be distinguished in this way are symbolized by jobs that each have specific tasks to fulfill which are written down in job descriptions (cf. Thiel and Mayer, 2009). Sustainable PA promotion in nursing home depends on whether the organization has assigned this task to a certain position in the staff appointment scheme. If this is not the case, it cannot be expected that nursing homes offer PA programs for the residents, not even if the executive and the nursing staff regard PA promotion as relevant.

Personnel decisions in organizations aim to allocate people to posts after the best possible suitability has been established (Luhmann, 2000). This requires specific criteria that guide the recruitment decision and (because recruitment alone does not guarantee a high degree of fitness between person and tasks) a permanent monitoring by the organization. Hence, personnel decisions should ensure that the recruited person fits with the organization-specific requirements and the requirements are not exposed to arbitrary individual influences. In our case, this means that even if PA promotion is anchored in the organization's program, an adequate offer will only be realized if the recruited staff is able to fulfill this task adequately. Conversely, people with competencies in PA promotion can compensate for the lack of corresponding structural guidelines, provided they have sufficient time.

Besides the formal structure of an organization, organizations are characterized by sets of *undecidable* decision premises, the so-called organizational culture (Luhmann, 2000). Undecidable decision premises are expressed as traditions, common values, and informal rules on how to deal with each other. Schein (2017) distinguishes three levels of organizational culture: (1) artifacts as visible and feelable phenomena, including the organizational *climate* with observable yet difficult to decipher behavior routines and rituals, (2) espoused beliefs and values (such as strategies and routines, philosophies and shared goals), and (3) basic assumptions and values that can be considered as an organization's informal code of conduct that determines behaviors and perceptions in an unconscious and taken-for-granted way.

The organizational culture is more or less a diffuse collective idea of what characterizes the organization at its core. Organization members take it for granted and seemingly understands it without having to be expressed (Luhmann, 2000 p. 243f.). The organizational culture, despite its indeterminacy, is highly binding, since it defines “the usual,” i.e., what is capable of consensus, referring to the history of the organization (Luhmann, 2000, p. 245). We assume that nursing homes that have the collective idea of being a healthy, activity-promoting organization will more likely attempt to promote PA during the lockdown.

Organizational culture is of particular importance as modern organizations, including nursing homes, have to deal with often contradictory requirements in their everyday lives in terms of maintaining organizational effectiveness and legitimacy. Organizations solve this problem by granting everyday operations a certain independency from the formal structure. In other words, while they legitimize organization operations through the formal structure, they carry out effective operations on the informal level independently of the formal structure (Dimaggio and Powell, 1983; Colignon, 2007). Due to the fact that the COVID-19 pandemic caused change effects in organizations, our focus will also be on the process of organizational change. A classic model for the explanation of organizational change was created by Lewin (1951). In his three-stage model of organizational change, he differentiates between unfreezing, transition, and refreezing. At the first stage, organizations tend to be unaware of impacts or the need for change. At the second stage, organizations tend to challenge the status quo and realize the need for change. At the third and final stage, organizations sustain changes and integrate new values, beliefs, and practices, also reflected in an adaption of structures.

In this paper, we aim to address the following main questions:

(1) How do nursing homes react to COVID-19-related restrictions?

With this question, we want to find out to which extent the Corona pandemic leads to an organizational change.

(2) How has PA promotion changed during the pandemic?

Thereby, we want to analyze the handling of COVID-19-related restrictions with regard to PA from the perspective of organizational sociology. This question comprises three subquestions:

(a) To what extent is PA promotion an organizational goal in nursing homes and how is PA affected by COVID-19-related restrictions?

In this regard, we ask whether PA-related organizational goals exist and, if yes, what relevance they have within the purpose programs of the nursing homes. Thereby, we also want to figure out whether PA promotion is part of the organization culture in nursing homes.

(b) How do the executive and nursing staff perceive PA-related impacts due to the COVID-19 pandemic, and how do they react to these impacts?

With this question, we want to figure out whether nursing homes have PA-related conditional programs to react to organizational restrictions or to what extent the response to such restrictions depends on the initiative of individual members of the organization only.

(c) Who is responsible for organizing PA activities during the COVID-19 pandemic?

This question focuses on the attribution of PA promotion to positions *via* communication means. Furthermore, we analyze to which extent the personnel can compensate a potential lack of structural guidelines for the promotion of PA. In this regard, we are also interested in whether caretakers are aware that inactivity can cause severe health-related problems for residents.

## Methods

### Setting

This study took place within a larger project, the Verhältnisorientierte Bewegungsförderung und individuelle Bewegungsberatung im Setting ĆAltenwohnheim‘ – ein biopsychosoziales Analyse– und Beratungsprojekt (BaSAlt) study on PA promotion and counseling in nursing homes (funded by the German Federal Ministry of Health 2019–2022, grant no. ZMVI1-2519FSB114). Data collection (document analysis, observations, photovoice, and interviews) had started 2 months before the COVID-19-related lockdown. After the nursing homes had been closed off, we adapted our study design to also cover the impact of the lockdown on PA in nursing homes.

Due to the exploratory nature of our study, we aimed to include a broad range of different nursing home settings. Thus, we used a maximum-variation sampling strategy regarding location, number of nursing places, and number of staff members, volunteers, and sponsors. Outpatient homes and geriatric rehabilitation centers were excluded. When selecting our final sample, we also attached importance to the nursing homes' stance on PA promotion. Here, we aimed to include both homes that regularly offered PA promotion programs before the pandemic and others that did not. During initial selection interviews, we discussed the conditions for participation in the project with the home management, which led to the exclusion of two homes due to time constraints during the COVID-19 pandemic.

The final sample consists of eight nursing homes in the Federal State of Baden-Württemberg in Germany. Nursing homes vary regarding their environmental contexts, responsible bodies, and organization forms, as well as capacity, and the composition of the resident populations. Three nursing homes are located in an urban area and five in more rural areas. We included homes from four different non-profit carriers. The participating nursing homes provide between 33 and 59 residents' places. The number of residents with a maximum level of care needs that equals 4 in the German healthcare system varies between 13 and 31 in the participating nursing homes.

Ethical approval for the study was granted by the Ethics Committee of the Faculty of Economics and Social Sciences at Eberhard Karls University Tübingen (no. AZ A2.5.4-096_aa). The Ethics Committee granted an amendment to acknowledge adaptations of the study design (assessment procedure and instruments, inclusion of digital elements in assessments and counseling, safety measures to be taken to minimize the risk of COVID19-spreading).

The collection and storage of personal data takes place in accordance with the European Data Protection Basic Regulation (DSGVO) and in coordination with the data protection officers of the institutions involved. Data is treated confidentially and processed pseudonymously. Prior to the COVID-19-related lockdown, home managers and staff were informed about the study. All participants gave written informed consent to participate in the study.

### Data Sources

#### Interview Data

In the first week of the lockdown, we informed the nursing home management that we would like to contact them monthly *via* telephone to assess the current status at their nursing home. The timing of these telephone interviews was adapted to relevant changes in the regulations of the country and state. In these semistructured interviews, we focused on COVID-19-related changes in the daily routines of nursing home staff and residents, the number of people suffering from an infection, the availability of protective equipment such as masks, and the general mood among staff members and residents. A semistructured interview guide was developed in such a way that COVID-19-related restrictions and their effects on nursing homes could be evaluated from the home administrators' perspectives. During the telephone interviews, the interviewer wrote down the answers of the home administrator in bullet points. Additional notes were supplemented from memory, immediately after the interview.

We conducted semistructured telephone-based qualitative interviews with *N* = 12 executive staff (nursing home managers, care services managers). We interviewed *N* = 10 female and *N* = 2 male executive staff, with professional backgrounds in nursing, social work, and rehabilitation therapy. Their mean length of occupation in this position was 3.5 years, with a range of 1 to 10 years. Their mean age was 42 years ranging from 31 to 54 years. Each executive staff completed at least one semistructured telephone interview between March and June 2020. Interviews lasted between 10 and 25 min. DA conducted all interviews. She is a sport scientist with expertise in PA promotion in aging populations and had established trust and relationships with the staff of participating nursing homes. She had presented the overall BaSAlt study and the goals prior to COVID-19-related restrictions and had conducted, among other data collection, systematic observations in the nursing homes.

#### Open-Ended Surveys

We developed an open-ended written survey to include the perspectives of staff, relatives/significant others, and external service providers. For staff, the survey covered aspects such as COVID-19-related restrictions on work, changes in daily routines, and the implementation of the contact restrictions issued by the BMG. With relatives of residents and external service providers, we mostly focused on the issue of contact restrictions. Eight weeks after the COVID-19-related lockdown, we sent the open-ended survey in paper-pencil format to three nursing homes that had agreed to participate in this data collection. A total of 66 surveys were handed out, 44 were returned to us, 24 filled out by staff, and 20 surveys by external partners (significant others/relatives, external service providers).

#### Documents

During the first week of the lockdown, we asked the home management for documents that describe purpose and conditional programs of the nursing homes, such as care concepts and mission statements. In order to figure out the attribution of tasks to positions *via* communication means, we also asked for management plans and weekly activity plans of the nursing homes. The weekly activity plans included information on how often homes offered PA such as fall prevention or stool gymnastics. By comparing weekly activity plans before the lockdown with the information obtained during the lockdown, we were able to assess the impact of COVID-19 on PA opportunities for home residents.

### Data Analysis

We analyzed the data consisting of written materials from the telephone interviews, answers from the open-ended written surveys, and documents with a reflexive thematic analysis approach (Braun and Clarke, 2006, 2019). We started data analysis with the MAXQDA software (version 2018.2) already during data collection to identify new angles as well as unclear issues and adapted subsequent interviews accordingly.

We employed both a deductive and inductive approach to data analysis. DA (background in PA promotion in aging populations) mainly coded the data. AF and AT (backgrounds in organizational sociology and health promoting settings) conducted the analysis, together with DA. Deductively, we used an organizational theory perspective to analyze how nursing homes dealt with the impact of COVID-19-related restrictions. To assess the role PA promotion played in the homes before the pandemic, we screened the general documents of the homes regarding decision programs (i.e., care concepts, mission statements) and the premises regarding the specification and distribution of organizational task, the so-called communication structures (management plans, and weekly activity plans of the nursing homes). For this purpose, we applied a keyword search, using terms related to PA (*e.g*., movement, activity, mobilization, fall prevention, stool gymnastics etc.).

Inductive data analysis of the interview materials involved repeated reading of the material to obtain an overall picture. In a subsequent step, the data was coded, and potential themes were developed. These theme candidates were then checked in relation to the coded extracts and the overall data set (Braun and Clarke, 2006). This stage in the analytic process was followed by a preliminary definition of themes and subthemes based on analyses and interpretations of the entire data set.

In a subsequent step, the data was coded and potential themes were developed. These theme candidates were then checked in relation to the coded extracts and the overall data set. We used multiple triangulation (regarding data sources, time, space or concrete case, and persons) to allow us to create a thick description and establish trustworthiness and rigor of findings (Smith and Mcgannon, 2018). To increase rigor, we held repeated peer debriefings among DA, AF, and AT, acting as critical friends (Smith and Mcgannon, 2018), as well as with other coauthors to discuss analysis, preliminary themes, and interpretation of themes.

For interpretation of themes, we conducted both a descriptive manifest and a more comprehensive latent analysis (Graneheim and Lundman, 2004; Graneheim et al., 2017), the latter to seek patterns that could explain the statements and observations from an organizational theory perspective.

## Results

In the following sections, we first give an overview on the stages of COVID-19 restriction measures. In a second step, we will present data on the question to which extent the impact of COVID-19 on PA promotion is manageable against the background of given organizational structures. In a third step, we analyze which role the organizational culture plays in the management of COVID-19 effects on PA promotion. Thereby, we also present which strategies to counteract the impact of COVID-19-related restrictions on PA promotion do executive and nursing staff consider particularly promising.

### Organizational Stages in Consequence of COVID-19-Related Restrictions

Against the background of Lewin's model of organizational change (Lewin, 1951), we identified three stages of COVID-19-related changes in nursing homes that impacted PA promotion: (1) external closure and search for emergency control, (2) organizational adaptations to create a livable daily life in the internal environment, and (3) slow reintegration of interactions with the external organizational environment.

#### Stage 1—External Closure and Search for Emergency Control

During the first stage of COVID-19-related restrictions, the Federal Ministry of Health and the state-level health authorities issued a complete ban of access to nursing homes, except for medical emergencies. This ban had a great impact on PA promotion. Due to the restrictions, volunteers and external service providers, such as physical therapists or trainers, were no longer granted access to the nursing homes to provide their services.

Relatives and significant others were also no longer able to access the homes to meet residents. In order to stay in contact, they had to use phone calls, very limited options for video-based meetings, or window conversations if possible.

Concerning the internal environment, nursing homes aimed to adapt their routines to meet COVID-19-related regulations. Apart from shared meals, such as lunch and dinner, group activities were canceled or relocated to take place outside, on terraces, with distance control. In addition, when a resident developed COVID-19 typical symptoms, all residents were isolated and joint activities were canceled. Concerning group activities, most homes had delegated PA promotion to external service providers, which resulted in group activities to drop-out of the focus of attention and reorganization. Therefore, most PA-related opportunities were canceled during this stage.

#### Stage 2—Adaptations to Create a Livable Daily Life in the Internal Environment

During the second stage, residents were no longer allowed to leave the nursing homes. Group activities of any kind were prohibited. While the official restrictions by health authorities appeared to be stricter than in stage 1, the nursing homes became more active in adapting their daily routines to make the daily life more livable.

To make the daily life more livable again, nursing staff took over parts of the activities that had been offered by external service providers in the past. Executive staff reached out to significant others and the broader community to interact with residents *via* special events, such as balcony concerts and Easter greetings in various forms. While these attempts increased external social interactions of residents, the internal interactions were still limited: regulations concerning distance-keeping measures, the wearing of facemasks, and contact limits had to be strictly followed. In addition to that, residents' activity radius was further limited to their room surroundings, which led to both restlessness and sedentary behavior.

Residents who previously had taken active walks started to lose their physical condition and walking security. Residents with dementia increasingly become restless [staff 1, open-ended survey comment]

In response to the continuation of the lockdown, further tightening of the restrictions and—relating thereto—prolonged inactivity of residents had visible effects not only regarding mobility patterns but also concerning mood-related expressions among residents. Nursing homes reacted and adapted their hygiene concepts to allow PA within clearly defined contexts: activities were carried out at distance in the courtyard. At the same time, staff acknowledged that those activities would not meet the same intensity and quality as organized offers by external provides would.

*In the living areas, attempts are being made to realize PA offers at a distance, but even this is not satisfactory. The added value is questionable if things such as fall prevention and gymnastics had previously been possible and now only very light and limited activation is possible [executive staff 1, interview]*.

In addition to group-based activities, both nursing staff and executive staff went for individual walks with residents in the nursing homes' premises, particularly to meet the need to move among residents with dementia.

#### Stage 3—Reintegration of External Environment

In the third stage, restrictions started to be loosened, particularly regarding the interaction with the external environment. With the reintegration of the external environment into the internal organizational environment, nursing homes had to deal with multi-fold organizational and logistical challenges. Each visitor had to be screened and residents had to be brought to the designed meeting areas. Meeting areas had to be disinfected after each visit and contacts had to be traced.

*A little busy at the moment, because visits are allowed again, but with that also increased organizing effort. Scheduling, cleaning, resident transport everything takes place in the cafeteria [executive staff 2, interview]*.

Residents were again allowed to leave nursing homes but had to wear masks inside their nursing home in the following 2 weeks, which appeared to be particularly challenging for residents with cognitive impairment or respiratory illness. Visits were allowed again, though limited to certain areas within the nursing homes, with advance notice and in compliance with the hygiene concept. Consequently, visits of external service providers were permitted again. Service providers could offer group activities, in compliance with hygiene with regulations such as distancing, ventilation, and contact limitations. However, this opening did not extend to PA promotion. Physical activities such as outdoor walks with significant others or volunteers, for example, still could not be realized.

### Impact of COVID-19 on PA Promotion-Related Formal Organizational Structures

#### Lack of Formalization and Internal Delegation of PA Promotion

Document analysis revealed that PA promotion already before the COVID-19 pandemic had generally not been an element of decision programs, such as nursing care concepts and mission statements. While PA promotion also did not appear as an explicit task in internal staff work descriptions, it was mentioned in weekly activity plans. Here, mostly external service providers, such as physiotherapists or volunteers, offered group activities once or twice a week. In this sense, external providers functioned as proxies for internal positions. However, due to the fact that this assignment of PA-related tasks to external providers was not structurally anchored in decision programs, they were not sustainable but vulnerable to unforeseen events that would limit resources or, like in the case of the COVID-19 pandemic, would limit access to the homes.

Once the COVID-19 restrictions came into place, the external providers could not enter the nursing homes anymore. As a consequence, in the second stage of restrictions, nursing home staff started to take over PA promotion efforts.

I trust in staff's creativity. [nursing home manager1, interview]

However, in most nursing homes, staff did not have an official mandate to do so. The general practice was rather to hand PA promotion over to day-care workers and nursing staff, in the hope that they would resolve the matter to everyone's satisfaction.

Day care workers and nursing home staff are trying to buffer everything. [executive staff 3, interview]

The belief in staff to buffer competencies and apply creativity indicated that nursing home managers and executive staff though PA promotion to be a task that could be fulfilled even if there was a lack of qualifications. Accordingly, the mental models of the executive staff regarding the promotion PA could be considered the main reason why PA promotion was not structurally anchored in decision premises.

After a while, some nursing homes adapted their strategies. They tried to continue the pre-COVID-19 weekly activity plans and delegated its execution to nursing staff.

Group mentoring is taken over by staff. [nursing home management 2, interview]

We still try to keep residents active, in small groups of maximum 8 people or in individual activities seasons. [nursing home management 1, interview]

If activities are offered, then only in small groups and not across stations. [nursing home management 3, interview]

The handing-over of PA promotion, however, did not happen in a clearly specified manner. Rather, personal knowledge and engagement of the nursing staff to promote PA promotion appropriately, was considered a functional equivalent to professional expertise trust by the executive staff^2^. This belief gave rise to a number of challenges: first, given the high fluctuation in nursing home staff, a lack of structurally fixed assignment of tasks to positions might result in a lack of sustainability, particularly if new staff had no knowledge or clear order to buffer activities that were usually offered by external providers. Second, to rely on the nursing staff to solve the problem would come to its limits if mistakes or refusal to perform the duties revealed that staff did not have the necessary knowledge, creativity, or level of personal engagement to execute PA promotion in a form and quality as external providers would have done.

PA promotion during the lockdown was not only hindered by an unspecific transfer of PA promotion to positions that normally have other functions but also characterized by a lack of monitoring of execution. The executive staff were aware of the additional workload linked to this delegation. They perceived it to be manageable to keep the residents as active and mobile as possible, without controlling whether this hand-over was working or not.

Staff has to take on extra responsibilities, but it seems to work so far. [nursing home management 2, interview]

This lack of organizing and monitoring of PA does not mean that PA promotion was not considered relevant. On the contrary, in some nursing homes, executive staff took over PA-related tasks by taking residents for walks on the premises to relieve nursing staff from this extra workload. Over time, the focus on residents' needs and well-being became even more important, as shown by attempts of executive staff to shift the workload focus from documentation of residents' status to activities for residents' well-being. The fact that documentation was considered unimportant is further proof that the quality of the implementation of PA promotion is basically rather a random product than the result of organizational planning.

The well-being of the residents is our top priority. Documentation? Only what is important for the public health department. [home management 4, interview]

#### Sticking to Formalized Mealtimes and Set-Ups Provides Room for PA and Social Interaction

At the moment, group activities, such as lunches, are still possible, but most of the other activities are unfortunately canceled. [executive staff 1, interview]

For the executive staff, adherence to formalized mealtimes and set-ups provided a daily structure to allow social contacts despite regulations for physical distancing. In addition, shared meals offered one of the few opportunities for PA since lunches and dinners tended to be served in an area separated from residents' rooms. Hence, the residents were at least forced to leave their room and make their way to the dining rooms.

Daily structure will be as preserved as possible. Dinners will be held together. [nursing home management 4, interview]

The relevance of shared meals for both staff and residents became even more visible when this activity had to stop due to isolation measures to be taken in case of COVID-19-like symptoms. Staff reported that the isolation measures, following from such a suspicion, affected all residents, which turned out to be a multi-fold challenge, not only concerning food supply but also concerning the overall logistical workload.

Four days of complete isolation from all residents was the nightmare, both logistically and food-wise. [nursing home management 1, interview]

On the other hand, shared meals also provided room and time for other activities, such as mobilization or individual walks, to take place. Isolation also led to one-to-one activation in the residents' individual rooms. In this sense, isolation provided the chance to mobilize the residents inhouse just because many other activities, particularly group activities, were not possible.

In some cases, instead of group activations, there were one-on-one activations in residents' rooms. Apart from that, I didn't notice much. [staff 9, open-ended survey comment]

### Impact of COVID-19 on PA Promotion-Related Organizational Culture

#### Organizational Climate—the Relevance of Informal Services of the External Environment

Some visitors compared the COVID-19-related restrictions in nursing homes to an imprisonment, with all entailed consequences.

There is a lack of social contacts. The last straw to which the residents cling is no longer available. A condition that is no longer sustainable. A comparison with an old people's prison is not far off. [significant other 1, open-ended survey comment]

These pessimistic descriptions of the situation reflected that staff's efforts to make the nursing homes livable environments during the lockdown were not visible for residents' significant others. Instead, officially communicated COVID-19-related restrictions (*e.g*., prohibition of visits or limited time and numbers of contacts) suggested that the institutions were exclusively concerned with the prevention of infections and less with residents' psychological and social well-being. From an outsider perspective, this was obviously difficult to accept.

The situation is not satisfactory for the visitors. [significant other 2, open-ended survey comment]

Being no longer able to enter the homes, significant others perceived only very limited “snapshots” of the daily life inside the organization, because daily routines and additional efforts of the nursing staff to activate the residents could not be observed during quarantine. This was also the case during stage 3, when significant others were still exposed to strict controls and time limits. For example, residents had to be picked up and brought to their relatives by the nursing staff, which also made it impossible for visitors to gain an insight into everyday life within the nursing homes.

The significant others' perception of the organizational climate as an only functionally oriented system was strongly influenced by the idea that significant others were the residents' only social contacts. They did not perceive interactions between residents or with nursing staff as social contacts or those interactions were not at outsiders' center of attention during COVID-19-related restrictions.

It is filled with much sadness. Much contact is no longer possible. We miss our mother. It's almost impossible to know how she's doing. It's all about feeling and physical contact. [significant other 4, open-ended survey comment]

In this context, the rule to wear a mask often appeared to be perceived less as a medical necessity than as an amplifier to residents' isolation.

It is an adjustment, as social contacts are enormously limited. Wearing the protective mask is uncomfortable. As a single person, there is a sense of loneliness. [significant other 3, open-ended survey comment]

#### Organizational Climate—Immense Workload Must Be Managed by Informal Arrangements

Most executive and nursing staff believed their organization to be able to cope with the new situation by mobilizing additional efforts. However, staff also emphasized that the pandemic had created a whole series of new tasks that had not been foreseen in any way. From the perspective of the nursing staff, a fundamental problem was that the exclusion of visitors led to a shift of responsibility regarding the residents' well-being. Given the importance of providing emotional support to residents for their well-being, nursing staff stressed that they now had to take on this important task themselves.

We must “catch” the residents. [staff, open-ended survey comment]

In this regard, informal organizational routines were considered a relevant factor. According to the nursing staff, the organizational climate, typically is characterized by a constant lack of time. This was particularly noticeable in the time of the pandemic when additional burdens already resulted from the necessity to comply with hygiene regulations.

Work becomes more exhausting when wearing a mask, more breaks are needed. [staff 4, open-ended survey comment]

Additional workload caused by room isolation of the residents. [staff 5, open-ended survey comment]

Increased workload due to compliance with hygiene regulations. [staff 6, open-ended survey comment]

Lack of time affected all areas of responsibility in nursing homes during the COVID-19 pandemic: care work for residents, group activities that had to be taken over from external providers, the consideration of COVID-19-related regulations in their work, and juggling work-life balance due to consequences of COVID-19 restrictions, such as home-schooling duties and childcare responsibilities in times of facility closure. A staff member summed up the time-responsibility dilemma during the lockdown very succinctly with the following statement:

*Little time* → *take care of many residents. [staff 3, open-ended survey comment]*

A basic problem was, according to the nursing staff, that the additional work caused by the pandemic had to be dealt with on the basis of already scarce existing time resources. This was attributed to a considerable extent to given organizational routines, which did not change through COVID-19-related restrictions but remained as (bad as) before.

Time management has not changed due to Corona, it is as bad as before. [staff 8, open-ended survey comment]

The statements of the nursing staff indicated that the “weaknesses of formalized decision premises could not be absorbed by informal arrangements.” Considering that the division of labor and the delegation of tasks had already been underdeveloped before the pandemic, the employees were forced to improvisatory solve the newly emerging tasks. This was also true regarding the integration of PA into the daily routine.

#### Organizational Climate—Mobilization Is Key, PA Not So Much

The PA-related organizational climate, as part of the culture of the organization, mirrored a diffuse collective idea of the importance of PA within the nursing homes. Our analyses show that PA was, in principle, regarded as very important for the residents' well-being. Thereby, staff were less concerned about the biological health of the residents than about the psychosocial strains that went along with the COVID-19-related isolation.

(We) started going outside this week with individual residents to avoid camp fever, wearing masks and so on. [staff 2, interview]

Apart of this, PA promotion equated individual mobilization only.

In some cases, instead of group activations, there were one-on-one activations in residents' rooms. Apart from that, I didn't notice much. [staff 9, open-ended survey comment]

In this context, the lack of formal regulations for PA promotion became obvious once again. By entrusting PA promotion solely to the nursing staff without saying exactly what should be done, any activation was largely limited to aspects that were considered important under the terms of the training of nurses. Individual mobilization was a basic part of the nurses' education and training and was accordingly described as one of the main foci during their daily care routines. Mobilization usually encompassed activities such as getting residents out of bed daily and promoting daily activities, such as tooth-brushing. For the nursing staff, PA promotion during the pandemic primarily was to integrate elements of physical therapy into mobilization routines.

However, some elements of individual mobilization, particularly outdoor walks, were used to substitute missing physical elements of patient-centered care. Patient-centered care was considered a core organizational goal in some nursing homes. According to nursing staff, these informal substitutes seemed to make closeness and physical contact possible. However, the replacement of lost physical interaction only partially was feasible due to the regulations on physical distancing.

Furthermore, outdoor walks are again possible, with a relative at a distance and a nurse. On the terrace in the garden, contact with relatives has been made all the time again. Nevertheless, the kind of volunteers and the usual closeness with hugging is missing. Not only for the residents also for the staff. [nursing home management 5, interview]

Taken together, staff and significant others mentioned several times that the potentially positive benefits of broader PA promotion at physiological, psychological, and social levels of health and well-being. With regard to the pandemic, they also highlighted examples of negative effects associated with current levels of inactivity in the nursing homes, such as physical decline, fatigue, mood swings, or depression.

Physical decline with increased risk of falling. [staff 11, open-end survey comment]

Very, very much, the residents are extremely tired, after small activations they already say they are tired. Their movements are massively restricted. [staff 10, open-ended survey comment]

In some cases, after the long quarantine period, residents are somewhat more ‘immobile’, not only physically. So much that worked before Corona does no longer work or only to a limited extent. [significant other 5, open-ended survey comment]

I can observe a mental and physical breakdown process in my mother. She moves much slower and her thinking and speech performance has decreased. The lack of closeness and physical contact, as well as the activity, she has stopped. It paralyzes her joy of life and leads to depressive attacks. [significant other 6, open-ended survey comment]

Residents' physical activity radius is much smaller than normal. This leads to more interaction between dementia patients, which does not lead to any positive interactions, but rather to cabin fever. [executive staff 1, interview]

However, this knowledge of the need for and benefits of PA promotion did not automatically lead to a change of nursing staff's code of conduct. Rather than explicitly incorporating PA promotion as part of the nurses' formal routines, both executive and nursing staff solely externalized this task to external service providers, leaving open the time, scope, and frequency of such measures.

It's now possible that the physiotherapist will come to our home. That means there are sports/activities twice a week. [nursing home management 5, interview]

## Discussion

### Organizational Coping With the COVID-19 Pandemic

Our study analyzed how COVID-19 restrictions impacted PA-related organizational structures in a selected sample of nursing homes in Germany. We identified three different stages in which the staff had to deal with pandemic-related restrictions. These stages cover external closure and search for emergency control, internal adaptation to make daily life livable in the internal environment and, later on, adaptations to reintegrate the external environment.

At first glance, our findings suggest that the reactions of staff members to COVID-19 restrictions follow Lewin's stage model of organizational change (Lewin, 1951). During the first stage, nursing homes seemed to be very insecure in how to address COVID-19 restrictions, unaware of potential progress toward change or counteracting impacts. During the second stage, nursing homes responded to make the internal environment livable again by buffering the external contacts and activities that had been lost due to restrictions. During the third and final stage of organizational change, some changes within staff's mental models could be observed, for example, with regard to the relevance of PA for residents' biopsychosocial well-being. This phase is still in development, given the continuously changing challenges of the ongoing pandemic.

From the perspective of our organizational sociological framework (Luhmann, 2000; Thiel and Meier, 2004; Rütten et al., 2009; Thiel and Mayer, 2009), however, nursing homes could not sustainably respond to the Corona pandemic in the matter of PA promotion due to their structural conditions. During the lockdown, nursing homes were particularly challenged with contradictory requirements in their daily routines in terms of maintaining organizational effectiveness and legitimacy (cf. Dimaggio and Powell, 1983; Colignon, 2007). Nursing home do not only lack PA-related decision programs but also appropriate communication means. PA promotion only is a marginal element of organizational routines. Since PA promotion is not explicit part of purpose programs, such as mission statements part and care concepts, PA at best is mostly a side-effect of activities that have different goals. Since the pandemic was unforeseeable, there had also been no conditional programs on how to compensate for activities “in motion” that would be eliminated by the lockdown. The analysis also shows that given communication means in nursing homes, such as management plans and weekly activity plans, had even before the pandemic not included the formalization and delegation of tasks related to the promotion of PA. Herein, our findings are in line with other research on effectively managed information and communication as decisive organizational factors to implementing change in healthcare settings (Li et al., 2018).

The lacking division of labor regarding PA promotion therefore resulted in an immediate stop of PA-related activities. The attempts to reorganize PA promotion were accordingly slow. Finally, typical personnel decisions in nursing home primarily focused on the aspect of patient care. This is understandable, as it cannot be expected that nursing staff are educated in terms of residents' appropriate physical activation. Accordingly, tasks related to PA promotion are normally outsourced to external providers and could not be entirely substituted by the nursing staff during the lockdown.

Our analyses of the organizational culture in nursing homes showed that the organizational climate in the institutions studied is characterized by an informal demand for patients' mobilization and social integration. However, it turned out that the understanding of mobilization was less that of “getting the patient moving,” but rather moving the (rather passive) patient around in a one-to-one interaction between caretakers and residents. Due to the collective belief that there was a lack of time resources in practically every respect, the organizational climate in the nursing homes was not favorable to broader PA promotion, particularly against the background of restrictions linked to the COVID-19 pandemic. Our findings reflect those of other studies in this respect. Heavy workloads, insufficient staffing on care routines, and lack of dedicating staff to implement specific activities, decrease the chances of those activities to be performed (Li et al., 2018).

The nursing homes tried to solve these challenges by carrying out effective operations on the informal level independently of the formal structure. Nursing staff took over responsibility for physically activating residents because they realized negative effects of physical inactivity, but this assumption of responsibility came about of the discretion of the staff members themselves. Those staff members acted as “champions” within their organizations (Li et al., 2018). Hence, nursing homes reorganized work and informally delegated PA-related tasks to nursing staff to counteract perceived negative effects of physical inactivity among residents. Nonetheless, the executive staff were aware that those attempts could not meet the amount, intensity, and quality of PA that had been offered by external providers prior to the COVID-19 pandemic.

Contribution to the Research on the Impact of COVID-19 on PA in Nursing Homes Given the acuteness of this study, the body of research that already has addressed COVID-19 impacts on nursing homes from an organizational sociological perspective is very limited; this is even more true when it comes to impacts on PA promotion in nursing homes. However, our findings appear to echo studies' findings on nursing care systems during COVID-19 which were based on a different theoretical perspective. Hower et al., for example, found out that the German nursing care system had already been at the limit of its capacity prior to the pandemic and that COVID-19 posed an additional challenge and burden on the system (Hower et al., 2020). They concluded that the demand posed on nursing staff for compensation of Corona-related restrictions went beyond physical care, but also included tasks such as quality control and documentation, compensation of staff shortage, or reorganization of social contacts and interactions with significant others *via* videoconferences or window visits (Hower et al., 2020). At the same time, their study came to similar conclusions as ours, namely that the personnel radiated a lot of optimism with regard to their coping abilities with the dynamically changing internal and external environments.

Our study did not explicitly consider the perspective of residents, apart from their function as service recipients. Nevertheless, our findings relate to the results of other studies that included interviews with residents. For example, although nursing home can be considered the residents' “hub of life” (Fänge and Ivanoff, 2009), those residents who are affected by (in our case, Corona-related) restrictions and regulations do not have a stake in how to address or counteract the impacts of those restrictions, why being a nursing home resident often goes along with a loss of self-determination and agency (Goffman, 1961; Hauge and Heggen, 2008).

Since early summer 2020, some states in Germany, such as Baden-Württemberg, where our study took place, have already partly taken back contact restrictions. Visiting regulations in nursing homes have been eased in order to counteract social isolation. However, as shown in our study, these relaxations are accompanied by many uncertainties and do not necessarily follow elaborated conditional programs. On the contrary, additional hygiene measures are installed to prevent an increased risk of infection. The volatility of the pandemic makes it necessary to constantly weigh up risks of infection and social isolation—for residents, as well as for relatives and caregivers. Consequently, any opening results in additional burden at the organizational and logistical level. Against this background, it remains to be seen how long the executive and nursing staffs retain their belief in the organizational coping skills of the institutions we investigated.

### Study Strengths and Limitations

The following limitations and strengths should be considered when interpreting the results of this study. Our goal was to contribute to a more nuanced understanding and theory-based explanation how nursing homes cope with the impacts of COVID-19 restrictions on PA promotion within the institutions. Against the background of constantly changing conditions, the results should be regarded as time and context specific. In line with the theoretical perspective of this paper, we focused only on the organizational perspective. There had been two reasons for excluding the residents' voice. First of all, we did not get access to the residents during the lockdown; therefore, it was not manageable to interview them. The lockdown prevented on-site interviews; interviews by phone or video-conference formats were not feasible due to technical issues (lack of Internet access in the homes) or residents' conditions (hearing/cognitive impairments). Furthermore, residents represent, from an organizational sociological perspective, the internal environment of the organization. In other words, they are recipients of the organization's services and thus do not reflect the perspective of the organization itself. In principle, it would have been very interesting to examine the extent to which the needs of the residents correspond to the perception of their needs by the organizational roles. However, since—on the one hand—this question was not at the core of our analysis and—on the other hand—access to the residents was not possible anyway, we excluded this aspect from this study.

Residents' voice and agency will play an important role in the main BaSAlt study (Thiel and al., submitted). Thereby, we will conduct photovoice documentation and analysis as well as focus groups with residents to identify their perceptions of options for and barriers to PA promotion (Thiel, Sudeck, Nieß, Eschweiler, Altmeier and Haigis, submitted).

We are convinced that our findings provide important insights into how care institutions deal with unforeseen events that lead to a rescheduling of everyday routines. This is particularly true in view of the fact that that our findings are consistent across the researched homes. This enables us to identify strengths and weaknesses in organizational routines when it comes to PA promotion in general and under unforeseen circumstances.

A limitation of our study is that although we analyzed a theoretically sound sample of nursing homes, we could only research those which had agreed to participate in the main BaSAlt study on PA promotion in nursing homes prior to the COVID-19 pandemic. Thereby, a response rate of 2/3 for the distributed written surveys appears to be at a rather good level, especially given the challenges and restrictions linked to the COVID-19 pandemic. Nevertheless, the situation in the nursing homes investigated might be significantly different from those who had chosen not to apply to participate in the BaSAlt study. We assume that the nursing homes investigated have both a particularly motivated and well-organized personnel.

### Practical Implications

This study has implications for practice and policy. At practice level, executive staff in nursing homes should become aware that sustainable, effective PA promotion needs to be incorporated into organizational structures, such as mission statement, care concepts, job descriptions, and weekly activity plans. To be able to respond with unforeseen crises, such as the COVID-19 pandemic, nursing homes should also develop conditional programs, more precisely, “if-then guidelines,” that provide strategic orientation for the continuation of relevant practices in critical situations.

At a policy level, the study implicates a need to negotiate resources between care-centered and bureaucratic activities in nursing homes. Increasing organizational, logistical, and bureaucratical demands linked to a challenge such as the COVID-19 pandemic will ultimately lead to losses in care-centered activities if not compensated for. PA promotion, as a by-sided activity, will be among the first activities to be lost—even though in the long run it might result in deteriorating biopsychosocial effects.

## Data Availability Statement

The datasets generated for this study can be requested via e-mail to the corresponding author.

## Ethics Statement

Ethical approval for the study was granted by the Ethics Committee of the Faculty of Economicsand Social Sciences at Eberhard Karls University Tübingen (no. AZ A2.5.4-096_aa). The Ethics Committee granted an amendment to acknowledge adaptations of the study design (assessment procedure and instruments, inclusion of digital elements in assessments and counseling, safety measures to be taken to minimize the risk of COVID19-spreading). The collection and storage of personal data takes place in accordance with the European Data Protection Basic Regulation (DSGVO) and in coordination with the data protection officers of the institutions involved. Data is treated confidentially and processed pseudonymously. Prior to the COVID-19-related lockdown, home managers and staff were informed about the study. All participants gave written informed consent to participate in the study.

## Author Contributions

AT, GS, AN, GE, and AF contributed to the conception and design of the overall BaSAlt study. AF, DA, HGra, and AT contributed to the concept and design of the substudy for this paper. DA, HGro, and AF collected data. DA, AF, and AT analyzed data. AF and AT wrote the first draft of the manuscript. DA, HGro, and JJ wrote sections of the manuscript. All authors contributed to critical manuscript revision, read, and approved the submitted version.

## Conflict of Interest

The authors declare that the research was conducted in the absence of any commercial or financial relationships that could be construed as a potential conflict of interest.

## Footnotes

^1^In German, the original term is “Kommunikationswege,” which also could be translated as “communication channels” or “communication paths.” Since this term describes a vertical and horizontal network structure which attributes organizational tasks to positions, we think that the term “communication means” is less misleading.

^2^This is similar to the practice how tasks are assigned to persons in voluntary organizations (see Thiel and Mayer, 2009).
