# Supplementary material for: “I Trust in Staff's Creativity”—The Impact of COVID-19 Lockdowns on Physical Activity Promotion in Nursing Homes Through the Lenses of Organizational Sociology
Source: Front Sports Act Living. 2020 Oct 7;2:589214. doi: 10.3389/fspor.2020.589214 (PMC7739680; doi:10.3389/fspor.2020.589214)
Supplement: Supplementary file 1 [file Table_1.DOCX]

# Supplementary file 1. Anchor quotes from the interviews and open-ended survey comments for the study on the impact of COVID-19 on physical activity promotion in German (original language) and English translation

| German | English |
| --- | --- |
| Bewohner, die vorher aktiv spazieren gingen, verlieren die Kondition und Sicherheit demente Bewohner werden zunehmend unruhig | “Residents who previously had taken active walks started to lose their physical condition and walking security. Residents with dementia increasingly become restless” (staff 1, open-ended survey comment) |
| In den Wohnbereichen wird versucht im Abstand Bewegungsangebote zu realisieren, aber auch das ist nicht befriedigend und der Mehrwert ist laut Aussage fraglich, wenn vorher Sturzprävention und Gymnastik möglich war und nun nur noch leichte Aktivierung | “In the living areas, attempts are being made to realize PA offers at a distance, but even this is not satisfactory. The added value is questionable if things such as fall prevention and gymnastics had previously been possible and now only very light and limited activation is possible” (executive staff 1, interview). |
| Gut, bisschen stressig momentan, da wieder Besuche erlaubt sind, aber damit einher auch vermehrter Orgaaufwand geht. Terminierung, Reinigung, Bewohnertransport, findet alles in der Cafeteria statt | “a little busy at the moment, because visits are allowed again, but with that also increased organizing effort. Scheduling, cleaning, resident transport everything takes place in the cafeteria” (executive staff 2, interview) |
| (Heimleitung) setze auf Kreativität der Mitarbeitenden. | “I trust in staff’s creativity.” (nursing home manager1, interview) |
| Alltagsbetreuer und Pfleger versuchen alles zu puffern. | “Day care workers and nursing home staff are trying to buffer everything.” (executive staff 3, interview) |
| Gruppenbetreuung wird von Mitarbeitenden übernommen. | “Group mentoring is taken over by staff” (nursing home management 2, interview) |
| Bewohnende werden weiterhin versucht ,in Kleingruppe zu beschäftigen, maximal 8 Personen, oder aber in Einzelaktivitäten | “We still try to keep residents active, in small groups of maximum 8 people or in individual activities seasons” (nursing home management 1, interview). |
| Wenn Aktivitäten angeboten werden, dann nur noch in Kleingruppen und nicht stationsübergreifend. | “If activities are offered, then only in small groups and not across stations.” (nursing home management 3, interview) |
| Mitarbeitende müssen vermehrt auch zusätzliche Aufgaben übernehmen, scheint aber soweit zu funktionieren | “Staff has to take on extra responsibilities, but it seems to work so far.” (nursing home management 2, interview) |
| Es werden Prioritäten gesetzt. Sprich: bei der Dokumentation werden die Sachen, die für das Gesundheitsamt wichtig sind, ausgefüllt. Bewohner stehen an oberster Stelle und Dokumentation kann man auch mal Dokumentation sein lassen, interessiert auch keinen, wenn des man nicht gemacht wurde. | “The well-being of the residents is our top priority. Documentation? Only what is important for the public health department.” (home management 4, interview) |
| Momentan sind noch gemeinsame Aktivitäten wie Mittagessen möglich, aber Großteil der Aktivitäten fällt leider aus. | “At the moment, group activities, such as lunches, are still possible, but most of the other activities are unfortunately cancelled.” (executive staff 1, interview) |
| Tagesstruktur wird so gut es geht erhalten. Gemeinsame Essen finden statt | “Daily structure will be as preserved as possible. Dinners will be held together.” (nursing home management 4, interview) |
| 4 Tage komplette Isolation von allen BW war der Horror, sowohl logistisch als auch essenstechnisch. | “Four days of complete isolation from all residents was the nightmare, both logistically and food-wise.” (nursing home management 1, interview) |
| Teilweise gab es statt Gruppenaktivierungsprogramm 🡪 1-zu-1 Aktivierung im Bewohnerzimmer | “In some cases, instead of group activations, there were one-on-one activations in residents' rooms. Apart from that, I didn't notice much” (staff 9, open-ended survey comment) |
| Es ist eine zunehmende Vereinsamung festzustellen. Gespräche werden zunehmend schwieriger. Es fehlen jegliche sozialen Kontakte. Der letzte Strohhalm, an den die Bewohner sich klammern, entfällt. Ein Zustand, der so nicht weiter tragbar ist. Ein Vergleich mit einem Altersgefängnis ist nicht fern. | “There is a lack of social contacts. The last straw to which the residents cling is no longer available. A condition that is no longer sustainable. A comparison with an old people's prison is not far off.” (significant other 1, open-ended survey comment) |
| Situation ist für die Besucher nicht befriedigend. | “The situation is not satisfactory for the visitors” (significant other 2, open-ended survey comment) |
| Vn viel Trauer begleitet. Viel Kontakt nicht mehr möglich. Mutter fehlt. Man kann sich vom Befinden kein Bild machen. Es geht viel über Gefühl + Berührung. | “It is filled with much sadness. Much contact is no longer possible. We miss our mother. It's almost impossible to know how she's doing. It's all about feeling and physical contact.” (significant other 4, open-ended survey comment) |
| Eine Umstellung, da soziale Kontakte enorm eingeschränkt sind. Das Tragen der Schutzmaske ist unangenehm. Als Alleinstehender ist eine Vereinsamung da. | “It is an adjustment, as social contacts are enormously limited. Wearing the protective mask is uncomfortable. As a single person, there is a sense of loneliness.” (significant other 3, open-ended survey comment) |
| Wir müssen die Bewohner auffangen. | “We must ‘catch’ the residents” (staff, open-ended survey comment) |
| Die Arbeit wird mit Mundschutz anstrengender, es werden mehr Pausen gebraucht. | “Work becomes more exhausting when wearing a mask, more breaks are needed.” (staff 4, open-ended survey comment) |
| Mehrarbeit durch Zimmerisolation der Bewohner | “Additional workload caused by room isolation of the residents” (staff 5, open-ended survey comment) |
| Höherer Arbeitsaufwand durch Einhaltung der Hygienevorschriften | “Increased workload due to compliance with hygiene regulations” (staff 6, open-ended survey comment) |
| Wenig Zeit 🡪 viele Bewohner pflegen | Little time → take care of many residents (staff 3, open-ended survey comment) |
| Zeitmanagement hat sich durch Corona nicht verändert, ist genauso schlecht wie davor. | “Time management has not changed due to Corona, it is as bad as before” (staff 8, open-ended survey comment) |
| hat diese Woche angefangen mit Bewohnern einzeln nach draußen zu gehen, mit Maske usw., damit diese keine Lagerkoller bekommen. | “(We) started going outside this week with individual residents to avoid camp fever, wearing masks and so on” (staff 2, interview) |
| Teilweise gab es statt Gruppenaktivierung 🡪 1-zu-1 Aktivierungen im Bewohnerzimmer. Sonst habe ich wenig mitbekommen. | “In some cases, instead of group activations, there were one-on-one activations in residents' rooms. Apart from that, I didn't notice much” (staff 9, open-ended survey comment) |
| Des weiteren sind wieder Spaziergänge im Freien möglich, mit einem Angehörigen im Abstand und einer Pflegekraft. Auf der Terrasse im Garten wurden die ganze Zeit schon wieder Kontaktgestaltung mit Angehörigen betrieben. Trotzdem fehlt die Art der Freiwilligen und die gewohnte Nähe mit Umarmen. Nicht nur den Bewohnern auch den Mitarbeitern. | “Furthermore, outdoor walks are again possible, with a relative at a distance and a nurse. On the terrace in the garden, contact with relatives has been made all the time again. Nevertheless, the kind of volunteers and the usual closeness with hugging is missing. Not only for the residents also for the staff." (nursing home management 5, interview) |
| Körperlicher Abbau mit erhöhter Sturzneigung | “Physical decline with increased risk of falling.” (staff 11, open-end survey comment) |
| Sehr, sehr viel, die Bewohner sind extrem müde, bei kleinen Aktivierungen sagen sie schon, wir sind müde. Ihre Bewegungen sind massiv eingeschränkt. | “Very, very much, the residents are extremely tired, after small activations they already say they are tired. Their movements are massively restricted.” (staff 10, open-ended survey comment) |
| Z.T. sind Bewohner nach der langen Quarantänezeit etwas „unbeweglicher“, nicht nur körperlich. Was vor Corona klappte, funktioniert nicht oder nur eingeschränkt. | “In some cases, after the long quarantine period, residents are somewhat more ‘immobile’, not only physically. So much that worked before Corona does no longer work or only to a limited extent.” (significant other 5, open-ended survey comment) |
| Ich kann bei meiner Mutter einen geistigen und körperlichen Abbauprozess beobachten. Sie bewegt sich viel langsamer und ihre Denk und Sprachleistung hat nachgelassen. Die fehlende Nähe und körperliche Kontakte, sowie die eingestellte Aktivität lähmen die Lebensfreude und führen zu depressiven Schüben. | “I can observe a mental and physical breakdown process in my mother. She moves much slower and her thinking and speech performance has decreased. The lack of closeness and physical contact, as well as the activity, she has stopped. It paralyses her joy of life and leads to depressive attacks.” (significant other 6, open-ended survey comment) |
| Bewegungsradius der Bewohner ist sehr viel geringer als sonst. Führt dazu, dass sich gerade Demente mehr interagieren und dies führt nicht zu positiven Interaktionen, eher Lagerkoller. | “Residents’ physical activity radius is much smaller than normal. This leads to more interaction between dementia patients, which does not lead to any positive interactions, but rather to cabin fever.” (executive staff 1, interview) |
| Es ist wieder möglich, dass die Physio kommt. D.h. es finden 2x die Woche Sport/ Aktivitätsangebote statt. | "It's now possible that the physiotherapist will come to our home. That means there are sports/activities twice a week.” (nursing home management 5, interview) |
